# A Retrospective Clinical Study on Factors Influencing the Failure of NCCL Restorations

**DOI:** 10.1155/2022/8048265

**Published:** 2022-04-11

**Authors:** Wanchanok Saengnil, Munlika Anuntasainont, Natchalee Srimaneekarn, Vesna Miletic, Pong Pongprueksa

**Affiliations:** ^1^Department of Operative Dentistry and Endodontics, Faculty of Dentistry, Mahidol University, Bangkok, Thailand; ^2^Department of Anatomy, Faculty of Dentistry, Mahidol University, Bangkok, Thailand; ^3^School of Dentistry, Faculty of Medicine and Health, The University of Sydney, Sydney, Australia

## Abstract

**Objectives:**

This retrospective study evaluated the factors influencing the clinical failure of noncarious cervical lesion (NCCL) restorations.

**Methods:**

Patients were routinely treated by undergraduate or postgraduate students and randomly received a clinical recall evaluation. A retrospective study was performed with two experienced calibrated examiners to evaluate NCCL restorations, including the critical parameters of retention, caries, marginal discoloration, and marginal integrity. The factors related to the restoration included gender, age, arch site, tooth position, the presence of occlusal wear facets, caries risk, operator, adhesive strategy, and composite. The clinical failure comparison between the parameters and factors was performed using the binary logistic regression analysis.

**Results:**

A total of 460 cervical restorations from 96 patients were evaluated. The adhesive strategy and the presence of occlusal wear facets were the most important factors influencing the parameter failure. Therefore, the highest failure was marginal integrity, in which the gingival marginal integrity failure was 50.7%, and the occlusal marginal integrity failure was 42.4%.

**Conclusions:**

The main factors influencing clinical failure for partial loss, marginal discoloration, and marginal integrity were the adhesive strategy and the presence of occlusal wear facets. Therefore, marginal integrity was the most frequent failure parameter.

## 1. Introduction

The etiology of noncarious cervical lesions (NCCLs) is multifactorial in terms of origin (erosion, abrasion, and abfraction), often initiated by a single factor, but then accumulates severity through other factors [1]. A recently favorable treatment option for NCCLs includes the placement of an adhesive restoration [2, 3], which is associated with the long-term clinical success and failure of NCCL restoration, including the intrinsic factors such as patient (behavior) [4] and tooth (tooth type, position, and occlusion) [5, 6] and the extrinsic factors such as operator (experience) [7, 8] and material (adhesive) [2]. In addition, the extrinsic factors are the most consequential to clinical failure [7, 8].

Moreover, NCCLs are slowly progressive on the tooth microstructure, leading to hypermineralized or sclerotic dentin formation to protect the pulpal structure. This microstructure formation presents a higher mineral deposition compared with sound dentin [9]. Therefore, adhesion to hypermineralized dentin is less effective than adhesion to healthy dentin due to the presence of acid-resistant hypermineralized structures, and the obliteration of dentinal tubules prevents acidic penetration into the hybrid layer, thereby reducing the quantity and quality of the hybrid layer and resin tags [10]. However, the previous study showed that the bonding effectiveness does not depend on the hybrid layer thickness and the resin tag length [11]. In addition, clinical suggestions for restoration to improve the bonding effectiveness of sclerotic dentin include a roughened dentin surface with a diamond bur [3] or a phosphoric acid pretreatment on dentin [10]. Moreover, a recent study recommended that the removal of hypermineralized dentin was unnecessary, as this affected the chemical interaction between functional monomers (10-MDP) and the hypermineralized layer of dentin [12].

Regarding recent adhesive systems, dental adhesive systems are classified into two major mode systems: etch-and-rinse mode (E&R) and self-etching mode (SE) [13]. The best clinical effectiveness of adhesives for NCCL restoration was for 2-step self-etching (2-SE) followed by 3-step etch-and-rinse (3-E&R), 1-step self-etching (1-SE), and 2-step etch-and-rinse (2-E&R) [14]. In addition, the use of selective enamel etching with phosphoric acid prior to the application of a self-etching adhesive, the so-called “selective enamel etching technique,” was recommended for improving the efficiency of self-etching adhesives on enamel margins for long-term clinical success [2, 14, 15]. Nevertheless, a previous study showed that 2-E&R was a more sensitive technique than 2-SE adhesive for undergraduate students, [7], which is in contrast to a previous study, in which 1-SE was a more sensitive technique than 3-E&R adhesive for undergraduate students [8].

Therefore, this study's objective was to investigate the factors that influence the clinical failure of NCCL restorations in different parameters.

## 2. Materials and Methods

Patients were referred to the university clinic, for the treatment of NCCLs with composite, in which the lesions were routinely cleaned with pumice and standardly isolated with cervical retraction cord, cotton rolls, and saliva ejector. The adhesive was used strictly according to the manufacturer's instructions under the supervision of instructors. The patient commonly underwent a recall examination every year; therefore, a retrospective clinical randomized study was performed between May 2018 and October 2018. The center approved the clinical trial protocol of Ethical Reinforcement for Human Research (COA MU-DT/PY-IRB 2018/025.3004). Two experienced clinical examiners were calibrated to evaluate NCCL restoration using the modified USPHS criteria, including assessing retention, caries, marginal discoloration, and marginal integrity (Table 1). The interexaminer agreement was assessed using Cohen's kappa test at 0.85, which was performed by the naked eye and a dental explorer. In case of disagreement between the investigators, a consensus was reached by reexamination and discussion.

The inclusion criteria were as follows: (1) NCCL restoration involved the occlusal enamel margin and gingival dentin margin of the root surface, (2) the restoration cavosurface margin did not involve more than 50% of the tooth height to control the lesion size between the operators, and (3) the restoration did not involve mesial and distal surfaces. The exclusion criteria were as follows: (1) uncontrolled systemic disease, (2) a controlled systemic disease that was influencing salivary flow rate, (3) orthodontic appliance treatments, (4) abutment tooth for fixed or removable prostheses, and (5) the restoration included base or liner materials on the record.

The data were collected as possible prediction risk factors for a longevity restoration as follows: gender, age, arch site (upper or lower), tooth position (quadrant 1, 2, 3, or 4), tooth type (anterior, premolar, or molar), occlusal wear facet (present or absent), caries risk (high, moderate, or low), operator (undergraduate or postgraduate student), adhesive (strategy), and composite (types). In addition, the caries risk assessment was evaluated following the ADA guidelines for caries risk assessment [16].

The restoration received a score of Alfa, Bravo, or Charlie for the categories of the modified USPHS criteria as the parameters (restoration loss, partial retention loss, caries, marginal discoloration, and marginal integrity), [17, 18] as shown in Table 1; however, the surface texture and anatomic form were not verified in this study. When the restoration was a category Bravo or Charlie, the restoration was adjusted as a clinical failure. Failed restorations were managed for refurbishment, repair, or replacement following the clinical management guidelines, [19] and lifespan was defined as the period from the initial treatment date to the examination date.

The sample size was calculated with one population proportion for comparison with the reference value (two sided), [20] with a significance level of *α* = 0.05, a desired power of 90% at *β* = 0.10, a reference value of *p*_0_=0.90, and a proportion of *p*=0.94. Therefore, the minimum sample size (*n*) was 371 restorations.

The statistical comparison between the results of factors and clinical failure parameters was performed using the binary logistic regression analysis at a significance level of 5% (*p* < 0.05). Cohen's kappa statistic was used to test conformity between two examiners and the reliability of each examiner.

## 3. Results

Data were collected on 460 NCCL restorations from 96 patients, with 36.1% men and 63.9% women. The average patient age was 60.9 years old (37–83 years), and the average longevity restoration period was 38.3 months (1–10 years). The restorative teeth were distributed as 53% upper teeth, 47% lower teeth, 28.1% anterior teeth, and 71.9% posterior teeth. The use of adhesive was as follows: 40.2% 2-E&R (Adper Single Bond 2; 3M ESPE, Seefeld, Germany, Optibond Solo Plus; Kerr, Orange, CA, USA, and ExciTE F; Ivoclar-Vivadent, Schaan, Liechtenstein), 20.2% 2-SE adhesive (Clearfil SE bond; Kuraray Noritake, Tokyo, Japan), and 39.6% selective enamel etching (phosphoric acid conditioning at enamel prier the Clearfil SE bond application). The use of composite was as follows: 70.4% Filtek Z350 (3M ESPE, Seefeld, Germany), 26.1% Estelite Sigma Quick (Tokuyama, Tokyo, Japan), and 3.5% Filtek Z250 (3M ESPE, Seefeld, Germany). The results are presented in Tables 2 and 3. Cohen's kappa statistical analysis standardized for two examiners was 0.95 for interexaminer and 0.96 for intraexaminer. Therefore, the clinical failures of restoration loss, partial retention loss, marginal discoloration, and marginal integrity parameters were influenced by various factors, while no specific factor influenced the caries parameter failures.

The failure on restoration loss, indicating Charlie (*C*), directly referred to the effectiveness of the adhesive strategy, is presented in Table 2. The clinical failure parameter for caries is presented in Table 2, indicated in Charlie categories. Other clinical failure parameters, including partial retention loss, marginal discoloration, and marginal integrity, are presented in Tables 2 and 3, including Bravo and Charlie categories (*B* + *C*).

The clinical failure of restoration loss (*C*) was related to the presence of occlusal wear facets (*p*=0.041) and adhesive strategy 2-E&R (*p*=0.043), 2-SE (*p*=0.050), as listed in Table 2. The clinical failure on partial retention loss (*B* + *C*) was separately evaluated on failures at the occlusal and gingival margins. Occlusal partial retention loss was significantly affected by the adhesive strategy factors, 2-E&R at *p*=0.013 (Table 2). The gingival partial retention loss was related to the patient's gender (*p*=0.014) and the presence of occlusal wear facets (*p*=0.004), as listed in Table 2.

Marginal discoloration failure (*B* + *C*) was separately observed at the occlusal and gingival margins. The occlusal margin discoloration was related to the occlusal wear facet (*p*=0.047) and adhesive strategy factor 2-SE (*p* < 0.001). Gingival marginal discoloration failure was related to gender (*p*=0.047), presence of occlusal wear facets (*p*=0.002), and adhesive strategy 2-SE (*p*=0.008), as presented in Table 3.

Marginal integrity failure (*B* + *C*) was separately observed at the occlusal and gingival margins, which was the worst failure parameter related to other factors. Occlusal marginal integrity failure was found in 42.4% of the total restorations, while gingival marginal integrity failure was found in 50.7%. Occlusal marginal integrity failure was related to the adhesive strategy 2-E&R (*p*=0.028), 2-SE (*p* < 0.001), while gingival marginal integrity failure was related to gender (*p*=0.010), presence of wear facets (*p* < 0.001), high caries risk (*p*=0.035), moderate caries risk (*p*=0.001), and operator (*p*=0.001), as presented in Table 3.

## 4. Discussion

This retrospective randomized clinical study revealed the factors influencing the failure of NCCL restoration performed by undergraduate and postgraduate students. The adhesives included 2-E&R (Adper Single Bond 2; 3M ESPE, Optibond Solo Plus; Kerr, and ExciTE F; Ivoclar Vivadent) and 2-SE adhesive (Clearfil SE bond; Kuraray), and selective enamel etching was performed with 37% phosphoric acid at the enamel margin followed by Clearfil SE bond application to the entire cavity. The composites included two nanofilled composites (Filtek Z350; 3M ESPE and Estelite Sigma Quick; Tokuyama) and a microhybrid composite (Filtek Z250, 3M ESPE). Therefore, the retrospective clinical failure was influenced by several factors: gender, the presence of wear facets, caries risk, operator, and adhesive strategy.

This retrospective study revealed the failure of NCCL restorations that had been treated by several undergraduate and postgraduate students, representing the actual completion of NCCL restoration in general dental treatment. In addition, several operator treatments provide valuable information since the dentist's skill is an important factor in the clinical success of a restoration [7, 8]. Nevertheless, a retrospective study is limited, as it lacks information on the original lesion, particularly on the lesion depth and tooth conditions such as tooth sensitivity and mineralized dentin. In contrast, most prospective studies included only a few operators, [2, 7, 8, 15], which is not representative of dentists in general. Thus, a prospective study is better for controlling and obtaining more information on the original lesion conditions, material, and treatment procedure.

In the present study, the clinical failure was not influenced by intrinsic factors, including age, arch, tooth position, tooth type, and extrinsic factors, on the composite for any parameters (Tables 2 and 3). The tooth position on the arch (upper or lower), tooth position (Q1, Q2, Q3, or Q4), and tooth type (anterior, premolar, or molar) did not influence any failure parameters. These findings contrasted with a recent meta-analysis study [5], in which anterior NCCL restoration had a higher clinical success rate than posterior restoration. This study composites included two nanofilled composites at 96.5% and a microhybrid composite at 3.5%, which is a huge difference between composites. However, the composite factor was not influenced by any clinical failure parameters, which is consistent with a previous clinical study that hybrid and microfilled composites had the same clinical success [21]. Besides, composite swelling and wear resistance can be causes of marginal discoloration and loss of marginal integrity, [22] as composite swelling is related to the water sorption and water solubility, which consequently depend on the resin matrix and the monomer polymerization [22–24]. Thus, the physical and mechanical properties of recent nanocomposites, nanohybrid composites, and microhybrid composites are hardly different [25, 26].

The intrinsic factors influencing clinical failure included gender, the presence of occlusal wear facets, and caries risk, while the extrinsic factors included operator and adhesive strategy. Gender was related to most clinical failures (partial retention loss, marginal discoloration, and marginal integrity), particularly at the gingival margin, which might indicate generally better oral hygiene for women than for men.

Occlusal wear facet was an intrinsic factor associated with the most clinical failure within the restoration loss, partial retention loss, marginal discoloration, and marginal integrity, particularly at the gingival margin. The result corresponded to a previous study that found higher staining at the gingival margin with the occlusal wear facet appearance [27]. This observation could be due to that the presence of occlusal wear facets is related to the etiological progression of NCCLs, which leads to a higher incidence of lesion progression as high stress and strain forces are concentrated in the cervical area with the presence of occlusal wear facets [6, 28, 29].

Caries risk assessment was associated with gingival margin failure for marginal integrity (Table 3). High caries risk and moderate caries risk had significantly higher failure than low caries risk on marginal integrity at the gingival margin. Caries lesions usually accumulate at the gingival margin, causing more defects. Bacterial biofilms at the gingival margin produce acid to destroy the tooth structure and create a marginal gap [30], consequently creating a defect at the gingival margin of restoration.

The operator is an extrinsic factor and includes undergraduate and postgraduate students, representing the operator skill. In the present study, the operator skill influenced the marginal integrity at gingival margin failure. In addition, the data revealed that undergraduate students used 2-E&R (46.9%), selective enamel etching (30.8%), and 2-SE (22.3%), while postgraduate students preferred selective enamel etching (77%), 2-E&R (11.5%), and 2-SE (11.5%). Thus, 2-E&R adhesive was used by undergraduate students over postgraduate students. The previous study showed that 2-E&R adhesive was more prone to salivary contamination than the selective etching adhesive strategy at the gingival margin [31]. Moreover, postgraduate students have more experience managing moisture control, restorative material, and polishing skills than undergraduate students. This result followed a previous study that showed a higher success rate of restoration marginal integrity in experienced dentists than in dental students [7, 8].

The adhesive strategy was the most influential factor related to restorative failures except for caries in this study. Selective enamel etching was the most favorable adhesive strategy and had the significantly lowest clinical failure rates on various parameters. Selective etching failed less than 2-E&R in most parameters (restoration loss, partial retention loss, and marginal integrity), which can be the result of phosphoric acid etching on dentin for E&R adhesive compared to a less aggressive acidic functional monomer (10-MDP) for Clearfil SE bond priming on dentin [32]. The 10-MDP functional monomer chemically interacts with the calcium of hydroxyapatite on dentin, forming a 10-MDP-Ca salt, which has a structure with low water solubility. This stable structure is expected to contribute to the hybrid and adhesive layers, improving the clinical longevity of the adhesively bonded restoration [13]. Moreover, selective enamel etching was associated with less restorative failure than 2-SE adhesive in terms of restoration loss, marginal discoloration, and marginal integrity, particularly at the occlusal enamel margin, and was associated with phosphoric acid etching at the enamel margin. This result was in agreement with previous studies, in which selective enamel etching had minor positive effects on marginal discoloration and marginal integrity in a long-term study [2, 33]. In contrast, selective enamel etching and 2-SE adhesive were not significantly different in clinical performance in a short evaluation period [15, 34].

## 5. Conclusion

The clinical failure was influenced by gender, the presence of occlusal wear facets, caries risk, operator, and adhesive strategy, among which the presence of occlusal wear facets and adhesive strategy were more influential factors in clinical failure. Marginal integrity was the most frequent failure for NCCL restoration. Thus, extrinsic factors, including operator experience and additional etching on the enamel margin prior to a 2-step self-etching adhesive application, reduced the clinical failure of NCCL restoration on marginal integrity.

## Figures and Tables

**Table 1:** Key parameters determining the overall clinical success rate using modified USPHS criteria.

Retention	*A*	Good retention
	*B*	Partially dislodged restoration
	*C*	Totally dislodged restoration

Caries	*A*	No caries present
	*C*	Caries present

Marginal discoloration	*A*	No discoloration
	*B*	Discoloration without axial penetration, can be removed by polishing
	*C*	Discoloration with penetration in the pulpal direction, cannot be removed by polishing

Marginal integrity	*A*	The restoration appeared to adapt closely to the tooth along the periphery of restoration. An explorer does not catch when drawn across the margin
	*B*	Explorer penetrates, edge of the restoration does not adapt closely to the tooth structure, and dentin is not exposed
	*C*	Explorer penetrates the crevice, in which dentin is exposed

*A* = Alfa, *B* = Bravo, *C* = Charlie.

**Table 2:** Distribution of the restorations, restoration loss, partial retention loss, and caries failure related to various factors presented as percentages.

Factor		Teeth	Restoration loss (*C*)	*p* value	Partial retention loss (*B* + *C*)				Caries	*p* value
					Occlusal	*p* value	Gingival	*p* value		
Gender	Male	36.1	3.6	0.567	9.6	0.435	13.9	0.014^*∗*^	0.6	0.376
	Female	63.9	3.4		5.8		6.1		1.0	

Age	15–59 years	50.0	3.5	0.222	7.0	0.970	7.8	0.629	0.9	0.746
	≥60 years	50.0	3.5		7.4		10.0		0.9	

Arch	Upper	53.0	2.0	0.986	5.3	0.915	7.0	0.132	0.8	0.926
	Lower	47.0	5.1		9.3		11.1		0.9	

Position	Q1	28.0	2.3	0.758	7.0	0.586	6.2	0.577	1.6	0.981
	Q2	25.7	1.7	0.651	3.4	0.302	8.5	0.435	0	0.995
	Q3	23.5	5.6	0.774	7.4	0.230	7.4	0.065	0	0.996
	Q4	22.8	4.8	Ref.	11.4	Ref.	14.3	Ref.	1.9	Ref.

Tooth type	Anterior	28.1	6.2	0.599	8.5	0.202	10.9	0.227	1.6	0.996
	Premolar	54.1	2.0	0.547	5.6	0.127	7.2	0.136	0.8	0.996
	Molar	17.8	3.7	Ref.	9.8	Ref.	11.0	Ref.	0	Ref.

Occlusal wear facets	Yes	61.5	4.9	0.041^*∗*^	8.8	0.128	12.4	0.004^*∗*^	1.1	0.525
	No	38.5	1.1		4.5		3.4		0.6	

Caries risk	High	7.0	6.3	0.684	15.6	0.389	12.5	0.963	0	0.998
	Moderate	75.6	3.2	0.276	6.9	0.555	9.5	0.330	0.9	0.185
	Low	17.4	3.8	Ref.	5.0	Ref.	5.0	Ref.	1.3	Ref.

Operator	Undergraduate	81.1	4.0	0.498	8.3	0.117	9.9	0.122	0.8	0.597
	Postgraduate	18.9	1.1		2.3		4.6		1.1	

Adhesive strategy	2-step E&R	40.2	5.9	0.043^*∗*^	11.4	0.013^*∗*^	12.4	0.163	1.1	0.895
	2-step SE	20.2	4.3	0.050^*∗*^	8.6	0.059	7.5	0.705	0	0.996
	Selective etching	39.6	0.5	Ref.	2.2	Ref.	6.0	Ref.	1.1	Ref.

Composite	Filtek Z350	70.4	2.8	0.999	6.2	0.914	9.0	0.998	0.3	0.999
	Estelite sigma quick	26.1	5.8	0.998	10.0	0.620	10.0	0.998	2.5	0.998
	Filtek Z250	3.5	0	Ref.	6.3	Ref.	0	Ref.	0	Ref.

Overall	Total (number)	100	3.5		7.2		8.9		0.9	

^*∗*^Association between the factor of restoration and the parameter failures, retention, and caries (*p* value <0.05, binary logistic regression analysis).

**Table 3:** Distribution of the marginal discoloration and marginal integrity failure related to various factors presented as percentages.

Factor		Marginal discoloration (*B* + *C*)				Marginal integrity (*B* + *C*)			
		Occlusal	*p* value	Gingival	*p* value	Occlusal	*p* value	Gingival	*p* value
Gender	Male	15.1	0.402	15.1	0.047^*∗*^	47.6	0.208	59.6	0.010^*∗*^
	Female	16.3		7.5		39.5		45.6	

Age	15–59 years	16.1	0.599	9.6	0.801	38.7	0.054	48.3	0.984
	≥60 years	15.7		10.9		46.1		53.0	

Arch	Upper	14.3	0.539	7.8	0.166	43.9	0.697	49.6	0.420
	Lower	17.6		13.0		40.7		51.9	

Position	Q1	15.5	0.602	8.5	0.307	42.6	0.683	53.5	0.517
	Q2	13.6	0.771	7.6	0.455	45.8	0.532	44.1	0.217
	Q3	16.7	0.953	12.0	0.819	39.8	0.988	52.8	0.986
	Q4	18.1	Ref.	13.3	Ref.	41.0	Ref.	52.4	Ref.

Tooth type	Anterior	16.3	0.741	13.2	0.151	34.9	0.166	55.0	0.827
	Premolar	15.3	0.670	10.4	0.067	46.6	0.251	49.8	0.654
	Molar	17.1	Ref.	4.9	Ref	41.5	Ref.	46.3	Ref.

Occlusal wear facets	Yes	18.0	0.047^*∗*^	13.8	0.002^*∗*^	42.8	0.599	59.4	<0.001^*∗*^
	No	12.4		4.5		41.8		36.7	

Caries risk	High	25.0	0.055	25.0	0.137	59.4	0.089	65.6	0.035^*∗*^
	Moderate	16.7	0.094	10.5	0.295	40.5	0.957	53.7	0.001^*∗*^
	Low	8.8	Ref.	5.0	Ref.	42.4	Ref.	31.3	Ref.

Operator	Undergraduate	16.9	0.195	11.8	0.073	45.6	0.098	54.4	0.001^*∗*^
	Postgraduate	11.5		3.4		28.7		34.5	

Adhesive strategy	2-step E&R	16.2	0.207	13.5	0.140	46.5	0.028^*∗*^	57.8	0.186
	2-step SE	28.0	<0.001^*∗*^	14.0	0.008^*∗*^	58.1	<0.001^*∗*^	51.6	0.280
	Selective etching	9.3	Ref.	4.9	Ref.	30.2	Ref.	42.9	Ref.

Composite	Filtek Z350	13.3	0.242	9.0	0.476	43.2	0.764	49.1	0.639
	Estelite sigma quick	21.7	0.961	13.3	0.899	40.0	0.793	55.8	0.979
	Filtek Z250	25.0	Ref.	12.5	Ref.	43.8	Ref.	43.8	Ref.

Overall	Total	15.9		10.2		42.4		50.7	

^*∗*^Association between the factor of restoration and the parameter failures, marginal discoloration, and marginal integrity (*p* value <0.05, binary logistic regression analysis).

## Data Availability

The data used to support the findings of this study are available from the corresponding author upon request.

## Additional Points

*Clinical Relevance Statement*. Operator experience, adhesiveness, and the presence of occlusal wear facets influenced the clinical failure, particularly marginal integrity. The marginal integrity was the most frequent failure of NCCL restoration. Postgraduate student operator and phosphoric acid etching of the enamel prior to 2-step self-etching adhesive resulted in less clinical failure, besides the types of composite did not influence the clinical failure of NCCL restoration.
